# A livelihood intervention to improve economic and psychosocial well-being in rural Uganda: Longitudinal pilot study

**DOI:** 10.1080/17290376.2016.1230072

**Published:** 2016-09-13

**Authors:** Bernard Kakuhikire, Diego Suquillo, Elly Atuhumuza, Rumbidzai Mushavi, Jessica M. Perkins, Atheendar S. Venkataramani, Sheri D. Weiser, David R. Bangsberg, Alexander C. Tsai

**Affiliations:** ^a^ MBA, is Senior Lecturer and Director of the Institute of Management Sciences, Mbarara University of Science and Technology, Mbarara, Uganda; ^b^ MIB, is a resident tutor at Quincy House, Harvard College, Cambridge, MA, USA; ^c^ MSc, is a study coordinator at the Infectious Diseases Research Collaboration, Kampala, Uganda; ^d^ AB, is a student at Harvard Medical School, Boston, MA, USA; ^e^ PhD, MPH, is a postdoctoral research fellow in the Chester M. Pierce, MD Division of Global Psychiatry, Massachusetts General Hospital, Boston, Boston, MA, USA; ^f^ MD, PhD is Assistant Professor of Medicine at Harvard Medical School, Boston, MA, USA; ^g^ MD, MPH, is Associate Professor of Medicine, University of California at San Francisco, San Francisco, CA, USA; ^h^ MD, MPH, is Founding Dean, Oregon Health Sciences University-Portland State University School of Public Health, Portland, OR, USA; ^i^ MD, is Assistant Professor of Psychiatry at Harvard Medical School, Boston, MA, USA

**Keywords:** HIV, poverty, social stigma, Uganda, VIH, la pauvreté, stigmatisation sociale, Ouganda

## Abstract

HIV and poverty are inextricably intertwined in sub-Saharan Africa. Economic and livelihood intervention strategies have been suggested to help mitigate the adverse economic effects of HIV, but few intervention studies have focused specifically on HIV-positive persons. We conducted three pilot studies to assess a livelihood intervention consisting of an initial orientation and loan package of chickens and associated implements to create poultry microenterprises. We enrolled 15 HIV-positive and 22 HIV-negative participants and followed them for up to 18 months. Over the course of follow-up, participants achieved high chicken survival and loan repayment rates. Median monthly income increased, and severe food insecurity declined, although these changes were not statistically significant (*P*-values ranged from 0.11 to 0.68). In-depth interviews with a purposive sample of three HIV-positive participants identified a constellation of economic and psychosocial benefits, including improved social integration and reduced stigma.

## Résumé

VIH et pauvreté sont inextricablement liés en Afrique subsaharienne. Des stratégies d'interventions économiques et sur les moyens de subsistance ont été proposées pour contribuer à atténuer les effets économiques néfastes du VIH. Cependant, peu d’études concernant les interventions se sont concentrées spécifiquement sur les personnes séropositives. Nous avons mené trois études pilotes afin d'évaluer d'une intervention sur ces moyens de subsistance initialement constituée d'une trousse d’orientation et d’un programme de prêt de poulets et de matériel associé pour créer des micro-entreprises avicoles. Nous avons recruté 15 personnes atteintes du VIH et 22 autres non contaminées et les avons suivies pendant 18 mois, ou plus. Tout au long du processus, des taux élevés de survie des poulets et de remboursement des prêts ont été obtenus par les participants. Le revenu mensuel médian a augmenté, et la grave insécurité alimentaire s’est estompée, bien que ces changements n’étaient statistiquement pas significatifs (les valeurs-p ont varié de 0.11 à 0.68). Des entretiens approfondis, avec un échantillon raisonné de trois participants séropositifs, ont identifié une multitude d'avantages économiques et psychosociaux, y compris une meilleure intégration sociale et une réduction de la stigmatisation.

## Introduction

HIV and poverty are inextricably intertwined in sub-Saharan Africa. Poverty and food insecurity are well-known risk factors for HIV acquisition among women (Miller, Bangsberg, Tuller, Senkungu, Kawuma, Frongillo *et al.* 2011; Schoepf 1988; Weiser, Leiter, Bangsberg, Butler, Percy-de Korte, Hlanze *et al.* 2007). HIV infection in productive working-age adults leads to gradual debilitation, and the subsequent loss of economic productivity and increased caregiver burden on other household members have substantial adverse economic impacts for the entire household (Rugalema 2000; Yamano & Jayne 2004). Food insecurity and poverty, in turn, impair the ability of HIV-positive persons to successfully overcome geographic and transportation-related barriers to obtain HIV medications from centrally located clinics and successfully adhere to their treatment regimens and remain engaged in care (Lankowski, Siedner, Bangsberg & Tsai 2014; Siedner, Lankowski, Tsai, Muzoora, Martin, Hunt *et al.* 2013; Tuller, Bangsberg, Senkungu, Ware, Emenyonu & Weiser 2010; Weiser, Palar, Frongillo, Tsai, Kumbakumba, dePee *et al.* 2014; Weiser, Tuller, Frongillo, Senkungu, Mukiibi & Bangsberg 2010). With increased risks for secondary HIV transmission resulting from suboptimal adherence and engagement in care, this complex web of mutually reinforcing effects yields a vicious cycle of HIV acquisition, worsening poverty and food insecurity, and increasing HIV-related morbidity (Weiser, Young, Cohen, Kushel, Tsai, Tien *et al.* 2011).

With the increasing availability and scale-up of HIV treatment in sub-Saharan Africa, it has been expected that the economic and psychosocial benefits of HIV treatment would have a normalizing effect on the meaning of living with HIV. In a number of qualitative and epidemiological studies, HIV-positive persons initiating treatment have described a constellation of economic and psychosocial benefits, including greater economic productivity and self-efficacy, reduced stigma, and improved mood (Campbell, Skovdal, Madanhire, Mugurungi, Gregson & Nyamukapa 2011; Martinez, Tsai, Muzoora, Kembabazi, Weiser, Huang *et al.* 2014; Tsai, Bangsberg, Bwana, Haberer, Frongillo, Muzoora *et al.* 2013; Ware, Idoko, Kaaya, Biraro, Wyatt, Agbaji *et al.* 2009). However, the extent to which HIV treatment alone can completely reverse pre-treatment losses and bring about economic and psychosocial normalcy has been questioned (Datta & Njuguna 2008; Treves-Kagan, Steward, Ntswane, Haller, Gilvydis, Gulati *et al.* 2016; Tsai *et al.* 2013). HIV treatment scale-up has had a beneficial effect on reducing the stigma attached to HIV, but it has not been completely eliminated (Chan & Tsai 2016; Chan, Tsai & Siedner 2015; Chan, Weiser, Boum, Siedner, Mocello, Haberer *et al.* 2015).

A number of economic and livelihood intervention strategies have been suggested to help mitigate the adverse effects of HIV infection in sub-Saharan Africa. Given that more than three-quarters of the poor population lives in rural areas, these intervention strategies typically involve informal businesses, agriculture, or animal husbandry. Such livelihood interventions have been shown to be effective in general population samples of adults, suggesting that they could potentially help HIV-positive persons as well (Blattman, Fiala & Martinez 2014; Rawlins, Pimkina, Barrett, Pedersen & Wydick 2014). In a recently published paper, Tsai, Bangsberg & Weiser (2013) described a conceptual model elaborating the linkages between poverty and HIV stigma, theorizing that poverty alleviation could potentially reduce the stigma attached to HIV. However, few livelihood intervention studies have focused specifically on HIV-positive persons (Pandit, Sirotin, Tittle, Onjolo, Bukusi & Cohen 2010; Tsai, Hatcher, Bukusi, Weke, Lemus Hufstedler, Dworkin *et al.* 2016; Weiser, Bukusi, Steinfeld, Frongillo, Weke, Dworkin *et al.* 2015). This is a problematic gap in the literature because HIV-positive persons face unique health and psychosocial challenges that could condition the extent to which they may benefit from such interventions (Datta & Njuguna 2008). Therefore, the objectives of our longitudinal pilot study were: (1) to evaluate the extent to which a livelihood intervention improved economic well-being and food security among HIV-positive and HIV-negative persons in rural Uganda; and (2) to evaluate the extent to which the livelihood intervention also reduced HIV stigma and increased social integration.

## Methods

### Study population and design

#### Setting

Data for this study were drawn from three pilot study cohorts – each receiving a slightly different version of a livelihood intervention scheme – that enrolled a mix of HIV-positive and HIV-negative persons living in Mbarara District in rural Uganda. The purpose of these pilot studies was to assess the feasibility of a livelihood intervention scheme for HIV-positive persons, and we used successive pilot studies to explore the specific type of model that was most appropriate for this setting. Ultimately, we aimed to develop procedures that could be used in a fully powered randomized controlled trial that would permit more definitive causal inferences to be drawn.

In consultation with counselors and HIV-positive key informants from the Mbarara Immune Suppression Syndrome clinic, we considered a number of different animal husbandry and agricultural models. Given the relatively low land and capital barriers to entry, potential ease of asset divisibility, and high product demand in the local setting, we ultimately selected a livelihood intervention based on poultry microenterprise. Mbarara District is located approximately 260 km southwest of Kampala, the Ugandan capital city. Mbarara Town is the primary commercial hub for the district and was listed in the 2014 census as having a population of 195,013 (Uganda Bureau of Statistics 2014), and most residents of the district live in outlying rural areas where the local economy is largely based on subsistence agriculture. Nearly one-third of the population is estimated to be living on less than $1.25 per day (The World Bank 2011); there is a high prevalence of food and water insecurity both in the general population and among HIV-positive persons specifically (Remans, Pronyk, Fanzo, Chen, Palm, Nemser *et al.* 2011; Tsai, Bangsberg, Frongillo, Hunt, Muzoora, Martin *et al.* 2012; Tsai, Kakuhikire, Mushavi, Vořechovská, Perkins, McDonough *et al.* 2016). HIV remains highly stigmatized in this setting despite the increasing availability of effective treatment (Chan *et al.* 2015).

#### Enrollment and study procedures

Potential study participants were eligible for enrollment if they were aged 18 years or older, lived in a permanent residence within 20 km of Mbarara Town, possessed land or had access to land sufficient for sheltering chickens, and were willing to attend all intervention training sessions and receive in-home visitors for technical support. Eligibility was not conditional on HIV serostatus, so as to avoid building resentment toward HIV-positive persons in the local community. All pre-enrollment assessments and study interviews were conducted in the local language (Runyankore). Upon enrollment, study participants were seen at pre-intervention baseline for structured interviews to assess economic and psychosocial well-being. Subsequent interviews during the study period occurred according to the schedule of study visits as described below. There was no control group. Research assistants who spoke the local language conducted interviews in a private research office near the Mbarara Immune Suppression Syndrome Clinic (for HIV-positive participants) or in the field at an agreed-upon location of the study participant’s choosing (typically near their homes or at their workplaces). Consistent with local etiquette and custom, at the conclusion of each interview, participants were offered a nominal incentive for their time (e.g. 1 kg of sugar or a bar of soap).

#### Pilot 1

For pilot 1, a convenience sample of HIV-positive men and women was recruited from the Mbarara Immune Suppression Syndrome Clinic in July–August 2011, and each study participant was followed for 12 months. They were provided a standardized intervention consisting of an initial orientation and single loan package of raw materials, along with ongoing technical assistance, to create a poultry microenterprise. The raw materials included materials to construct a poultry shelter, related implements such as chicken feed, and 100 ‘layer’ chicks (i.e. female chickens raised primarily for egg production); ongoing technical assistance occurred through home visits every week for two months. The median value of the total package of raw materials was 1.3 million Ugandan Shillings (equivalent to 510 U.S. dollars, given the exchange rate at the time of enrollment; interquartile range [IQR], $448–531). A number of important barriers were encountered in pilot 1. Namely, study participants experienced some demotivation resulting from their having to care for the layer chicks for a full 19–23 weeks before egg production commenced (Byarugaba 2007). In addition, many felt overwhelmed by having to care for 100 chicks all at once.

#### Pilots 2 & 3

Convenience samples of both HIV-positive and HIV-negative men and women were recruited from the community into pilot 2 in February 2012 and into pilot 3 during October 2012–January 2013. Each participant was followed for 18 months. The standardized intervention was similarly oriented toward establishing a poultry farm and microenterprise, but several modifications were introduced due to difficulties encountered in the first pilot. To address concerns about the lengthy period of upkeep prior to revenue generation, we instead provided participants with ‘broiler’ chicks (i.e. male chickens raised primarily for meat production). To address concerns about the excessive upfront responsibility for upkeep, we introduced the broiler chicks in 3–4 loan cycles of gradually increasing size. The median value of the total package of raw materials given across all loan cycles was 1.3 million Ugandan Shillings (equivalent to 483 U.S. dollars; IQR, 430–506). Receipt of each successive loan was contingent upon repayment. In pilot 3, participants received the same intervention but were enrolled in groups of 4–5 members, and the group assumed joint liability for the loan.

Participants provided written informed consent, either with a signature or with a thumbprint. Ethical approval for all study procedures was obtained from the Partners Human Research Committee, Massachusetts General Hospital; and the Institutional Review Committee, Mbarara University of Science and Technology. Consistent with national guidelines, we received clearance for the study from the Uganda National Council for Science and Technology and from the Research Secretariat in the Office of the President.

### Data collection and analysis

The primary outcomes of interest were related to economic well-being and were all obtained by self-report. We surveyed participants about their monthly household income. Because educational expenditures comprise one of the largest line items in a rural Ugandan family’s budget, we also asked participants about the total quarterly amount spent on school fees among children in the household. Food insecurity was measured with the Household Food Insecurity Access Scale (Swindale & Bilinsky 2006), which has been validated and used extensively in this context (Tsai, Bangsberg, Emenyonu, Senkungu, Martin & Weiser 2011). In the baseline sample, the food insecurity scale had good internal consistency, with Cronbach’s alpha = 0.80 (95% confidence interval, 0.69–0.90). The recommended algorithm was used to identify participants meeting criteria for severe food insecurity. Due to an administrative error, the food insecurity scale was not administered in pilot 1.

Because there was no control group, we conducted ‘pre-post’ analyses only. Continuous outcome variables (household income, expenditures on school fees) were compared across study visits using a nonparametric test for trend across ordered groups (Cuzick 1985). The dichotomous outcome variable (severe food insecurity) was compared using Pearson’s chi-squared test. All analyses were conducted using the Stata statistical software package (version 13.1, StataCorp, College Station, Tex.).

Following completion of participation in the intervention study, we selected three HIV-positive study participants (one man and two women) for in-depth exit interviews. These participants were selected as a purposive sample, to ensure that diverse viewpoints were represented, with final selection based on interest and availability of time. The research assistants who conducted the in-depth interviews were blinded to the study participants’ survey responses. In-depth interviews consisted of in-person, semi-structured interviews designed to elicit information about how the participants experienced the intervention program and its impacts on various psychosocial dimensions of their lives. Interview probes were organized around their experience of the intervention itself (e.g. training, process, and repayment) as well as perceived economic and psychosocial changes attributable to the intervention. The participants’ open-ended responses were reviewed for common themes and then summarized.

## Results

### Quantitative findings

**Fig. 1.**
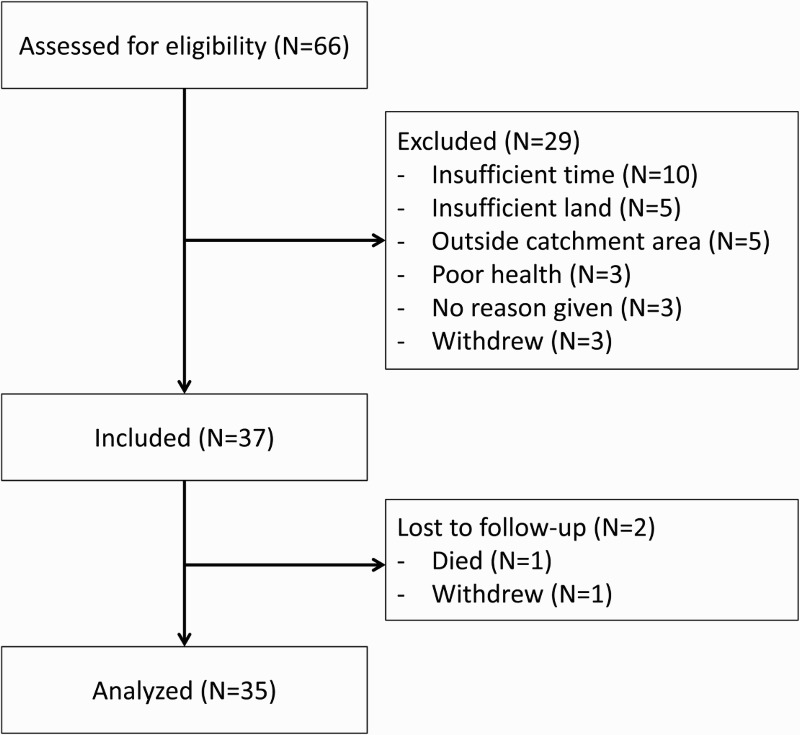
Flow diagram of participants assessed for eligibility and enrolled into the study.

Participant flow is shown in Fig. 1. Of 66 persons initially approached for enrolment across the three pilots, 5 were ineligible, as they were found to live outside of the catchment area, 5 did not have sufficient access to land, and 16 declined (10 did not believe that they would have enough time to devote to the project, three were in poor physical health, and three gave no reason). An additional three participants dropped out after enrollment but prior to baseline data collection, citing time, or family commitments. We included baseline data for a total of 37 participants: 11 participants in pilot 1 and 29 participants in pilots 2 and 3. Participants in pilot 1 were followed for a median of 365 days (interquartile range [IQR], 361–367); one participant died and was not re-interviewed at the 12-month follow-up. Participants in pilots 2 and 3 were followed for a median of 488 days (interquartile range [IQR], 452–520). At 12- and 18-month follow-up, 25 participants in pilots 2 and 3 were re-interviewed, with one participant who had withdrawn from pilot 3 prior to the 12-month follow-up.

Summary characteristics of the samples are described in Table 1. Most participants in the pilot 1 were women, as were most of the participants in pilots 2 and 3. Slightly more than one-third of the sample was HIV-positive. The majority of participants were engaged in farming occupations and sold some portion of their agricultural production for cash. Most participants used public water sources such as communal taps, open wells, or boreholes. All participants lived outside of Mbarara town.

**Table 1.** Baseline characteristics of participants enrolled in three pilot studies conducted in Mbarara, Uganda, 2011–2013 (*N* = 37).

	Median (interquartile range) or Number (percent)	
	Pilot 1 (*N* = 11)	Pilot 2 & 3 (*N* = 26)
Age, years	36 (35–41)	43 (34–49)
Female	10 (91)	20 (77)
HIV-positive	11 (100)	4 (15)
Farming occupation	5 (45)	19 (73)
Educational attainment		
None	0 (0)	6 (23)
Primary schooling only	4 (36)	9 (35)
Some secondary or greater	7 (64)	11 (42)
Number of children	4 (2–5)	5 (3–7)
Any agricultural production in household	9 (82)	23 (88)
Primary disposition of household agricultural production		
Consumed by household members	3 (33)	6 (26)
Some portion sold for cash	6 (66)	17 (74)^a^
Primary use of public water source	8 (73)	23 (92)^b^
Distance to water source, meters	250 (10–700)	350 (20–500)
Travel time to Mbarara town, minutes	40 (30–90)	30 (15–60)

^a^Number of missing observations = 3.

^b^Number of missing observations = 1.

Over the course of the study, chicken survival exceeded 90%. The median repayment rate was 59% in pilot 1 and 82% in pilots 2 and 3. No changes in the outcome variables were statistically significant (Table 2). Median monthly income increased from approximately 46 USD at baseline to 115 USD at 12-month follow-up in pilot 1 (*z* = 1.6, *P* = .11), and from 42 USD at baseline to 115 USD at 18-month follow-up in pilots 2 and 3 (*z* = 1.1, *P* = .26). Similarly, median quarterly expenditures on school fees increased. In pilots 2 and 3, the proportion with severe food insecurity declined from 48% to 36% (*χ*^2^ = 0.77, *P* = .68).

**Table 2.** Changes in outcome variables among participants enrolled in three pilot studies conducted in Mbarara, Uganda, 2011–2013 (*N* = 37)^a^.

	Baseline	Visit 2	Visit 3^b^	Test statistic (*P*-value)
*Pilot 1 (“layers)*				
Median monthly income^c^	46	58	115	*z* = 1.6 (*P* = .11)
Median quarterly expenditures on school fees^c^	84	130	194	*z* = 1.5 (*P* = .15)
*Pilots 2 & 3 (“broilers”)*				
Median monthly income^c^	42	48	115	*z* = 1.1 (*P* = .26)
Median quarterly expenditures on school fees^c^	89	115	115	*z* = 1.0 (*P* = .32)
Number of participants with severe food insecurity^d^	12 (48%)	10 (40%)	9 (36%)	*χ*^2^ = 0.77 (*P* = .68)

^a^In the first pilot, one participant died and was not re-interviewed at the 12-month follow-up. In the third pilot, one participant withdrew from the study prior to the 12-month follow-up.

^b^For participants in pilot 1, visit 2 occurred at 6 months of follow-up and visit 3 occurred at 12 months of follow-up. For participants in pilots 2 and 3, visit 2 occurred at 12 months of follow-up and visit 3 occurred at 18 months of follow-up.

^c^Amounts expressed in U.S. dollars (USD) converted from Ugandan Shillings (USh), using the exchange rate governing at the time of enrollment (2600 USh:1 USD for pilots 1 and 3; 2300 USh:1 USD for pilot 2).

^d^Due to an administrative error, the Household Food Insecurity Access Scale was not administered in pilot 1.

### Exit interviews

The three HIV-positive participants selected for the in-depth interviews were generally positive about the intervention and spontaneously enumerated several different types of benefits that they attributed to their participation. Representative quotations from these study participants are described below.

#### Economic benefits

All three participants selected for the in-depth interviews commented that they had received substantial economic benefits from participating in the intervention. These benefits were typically described in terms of new consumption expenditures. For example, all three participants remarked that they were now able to pay their children’s school fees. Two representative quotes are shown below:

The project has helped to lift my status in the village. I have transferred my children from the poor village schools to private schools. My children tell the other children that we eat eggs. Every weekend we have a routine of eating eggs. The children look better because they eat eggs at least once per week. People gossip that my children eat eggs every week. I think they may be jealous.

Before I had to look around for money on the first day of school. But now I can send my children to school on the first day when they have fully paid all the fees. Now there is a constant income, I can use the money to educate my children. Before I would constantly have to negotiate a schedule with the headmaster’s office.

#### Psychosocial benefits

All three participants commented that the intervention had contributed to social reintegration, status enhancement, and stigma reduction. Two representative quotes are shown below:

Formerly I had never seen anyone invite me to big functions because they didn’t think I could contribute anything. Recently there was a fundraising at the mosque, and I brought two trays of eggs. People give me respect. Now even at schools they are inviting me to attend meetings.

People now call me for important community meetings. People now pick me to talk, but before they would ignore me.

One participant explicitly linked these processes to the economic benefits of the intervention:

Before I was more needy. I felt like I would only tell people ‘I need salt’. Now that I earn funds, people who used to keep their distance are now coming back. Every day at least you have some money in your pocket. If you are short of money you can borrow from others because they know you have a source of funds and you will repay. Yesterday I was able to borrow 100,000 [Ugandan Shillings] from my friend.

One participant commented that engaging in productive activity had helped to reduce her symptoms of depression:

Now I feel more peaceful in the mind. Now when I see people I don’t imagine they are whispering about my condition. When you are just idle, the [HIV] disease will progress because you are thinking a lot about it. Now you have other things to attend to, how much water is there for the chickens. If you spend all your time thinking about the disease you feel worse.

## Discussion

In this small pilot study of a livelihood intervention for HIV-positive and HIV-negative men and women in rural Uganda, we report two primary findings. First, we observed preliminary evidence of benefit in the sustained increases in economic well-being and food security over the course of 12- to 18-month follow-up. Second, in-depth interviews revealed that the livelihood intervention can potentially reduce the stigma of HIV by providing HIV-positive persons with a means of economic and social integration. These findings have important programmatic implications for HIV-positive persons in rural areas of sub-Saharan Africa.

The large, sustained (albeit non-statistically significant) increases in household income observed among livelihood intervention participants in this study provide suggestive evidence of benefit. These observations are consistent with the findings of related studies showing beneficial economic impacts of microfinance loans on men with established businesses (de Mel, McKenzie & Woodruff 2012) and of unconditional cash transfers on informal businesses among both men and women (Blattman *et al.* 2014). A related literature has demonstrated economic benefits of child sponsorship programs (Wydick, Glewwe & Rutledge 2013) and livestock donation programs (Rawlins *et al.* 2014). While it remains an unresolved question as to whether microfinance loans or unconditional cash transfers are more cost effective after accounting for implementation-related costs, available evidence suggests that the choice of intervention depends largely on the context and objectives of the intervention (Hidrobo, Hoddinott, Peterman, Margolies & Moreira 2014; Hoddinott, Sandström & Upton 2014).

The psychosocial benefits of the livelihood intervention as described during the exit interviews were largely unanticipated at study initiation but emerged shortly after the first pilot was completed. However, the observed impacts of the livelihood intervention on social reintegration, status enhancement, and stigma reduction are consistent with a theory of stigma showing that an important driver of HIV stigma in sub-Saharan Africa is the widespread perception that HIV-positive persons are economically incapacitated and unable to make sustained reciprocal contributions within local solidarity networks (Tsai *et al.* 2013). Our findings are also consistent with a recently published study showing that an agricultural intervention reduced stigma and increased social integration for HIV-positive persons in rural Kenya (Tsai, Hatcher, Bukusi, Weke, Lemus Hufstedler, Dworkin *et al.* 2016). In many low-income countries in sub-Saharan Africa, where formal social protection schemes are either limited or absent, the norm of economic reciprocity serves to regulate risk-sharing and informal insurance (Fafchamps 1992). Theoretical models of stigma in evolutionary social psychology (Kurzban & Leary 2001; Neuberg, Smith & Asther 2000), and instrumentalist analyses of social capital (Portes & Sensenbrenner 1993), both predict the exclusion of persons who are perceived to violate social norms. This form of HIV stigma and social exclusion straddles the ‘symbolic’/‘instrumental’ distinction traditionally employed in the field: it is *symbolic,* in that it derives from the symbolic association between HIV, disability, and death; and it is also *instrumental,* in that it serves the instrumentalized purpose of excluding economically inadequate persons from the community. HIV-associated economic incapacity as a specific driver of HIV stigma has been described not only in Uganda but also in multiple other resource-limited settings (Bond 2006; Datta & Njuguna 2008; Maman, Abler, Parker, Lane, Chirowodza, Ntogwisangu *et al.* 2009; Maughan-Brown 2006; McGrath, Ankrah, Schumann, Nkumbi & Lubega 1993; Niehaus 2007; Tsai, Bangsberg & Weiser 2013). These descriptions are also consistent with qualitative research conducted in Uganda, Nigeria, and Tanzania by Ware *et al.* (2009), who concluded that potentially being excluded from an interdependent economy of mutual benefit is precisely why the stigma of HIV is so feared. Given that HIV continues to be highly stigmatized throughout sub-Saharan Africa, we believe that the use of livelihood interventions to reduce HIV stigma deserves further exploration.

Interpretation of our findings is subject to several important limitations. First, this study did not include a control group of participants who lacked access to the livelihood intervention. It is therefore possible that the improvements in economic and psychosocial well-being were driven principally by secular trends related to the time course of HIV treatment (Tsai *et al.* 2013; Venkataramani, Haberer, Thirumurthy, Boum, Siedner, Kembabazi *et al.* 2014). The participants may have also been engaged in other livelihood strategies. Second, the small sample size limited the extent to which definitive conclusions could be drawn. Third, we did not conduct a formal qualitative study. The themes about psychosocial benefits emerged spontaneously in the exit interviews and could have resulted from social desirability bias. These findings would need to be confirmed with either a larger qualitative sample, or an epidemiologic study in which instruments of good reliability and validity were administered to study participants over time. These psychosocial constructs could be measured with structured instruments such as have been developed to measure HIV stigma (Tsai, Weiser, Steward, Mukiibi, Kawuma, Kembabazi *et al.* 2013) or depression symptom severity (Tsai 2014). Fourth, while repayment rates exceeded those described in studies of similar economic interventions for HIV-positive persons (Pandit *et al.* 2010), they did not approach 100%. Mathematically, an economic intervention with a 0% repayment rate is equivalent to a donation. Therefore, while a less than 100% repayment rate might not necessarily be viewed as ‘sustainable,’ discussion of its place in health and development programming in resource-limited settings should acknowledge its value, that is, what is the *value* of an intervention that improves food security or reduces HIV stigma? Fifth, and finally, with the relatively short follow-up of 12–18-month follow-up period, we were unable to assess the impact of the intervention on long-term outcomes (de Mel *et al.* 2012).

Despite these limitations, we conclude that this livelihood intervention showed preliminary evidence of both economic and psychosocial benefits. Its potential impacts on HIV stigma may be most appealing, given the relative paucity of evidence supporting anti-stigma interventions in the literature (Stangl, Lloyd, Brady, Holland & Baral 2013). In the most recent Demographic and Health Surveys conducted throughout sub-Saharan Africa, negative attitudes toward HIV-positive persons remain highly prevalent (Chan & Tsai 2016; Chan *et al.* 2015; Tsai 2015). In Uganda, which also happens to be the only country in sub-Saharan Africa to have experienced an increasing incidence of HIV over the past decade, HIV has become even more stigmatized despite treatment scale-up (Chan *et al.* 2015). These population trends are worrisome because HIV stigma undermines uptake of HIV testing and increases HIV transmission risk behaviors in the general population (Delavande, Sampaio & Sood 2014; Kelly, Weiser & Tsai 2016). Among HIV-positive persons specifically, internalized stigma is associated with social isolation, lack of disclosure, depression, failure to link to care, and reduced treatment adherence (Govindasamy, Ford & Kranzer 2012; Katz, Ryu, Onuegbu, Psaros, Weiser, Bangsberg *et al.* 2013; Takada, Weiser, Kumbakumba, Muzoora, Martin, Hunt *et al.* 2014; Tsai *et al.* 2012; Tsai, Bangsberg, Kegeles, Katz, Haberer, Muzoora *et al.* 2013). Thus, despite major advances in understanding how effective treatment prevents secondary HIV transmission, the persisting stigma of HIV threatens to compromise treatment and prevention efforts. New interventions to reduce the stigma of HIV are urgently needed.
